# Supplementary material for: Predicting adherence to gamified cognitive training using early phase game performance data: Towards a just-in-time adherence promotion strategy
Source: PLoS One. 2024 Oct 2;19(10):e0311279. doi: 10.1371/journal.pone.0311279 (PMC11446454; doi:10.1371/journal.pone.0311279)

Supporting Information

S1 Fig. Evaluation of Game Performance Effects Using SHAP Values Summary Plot

(a) Minimal Adherence

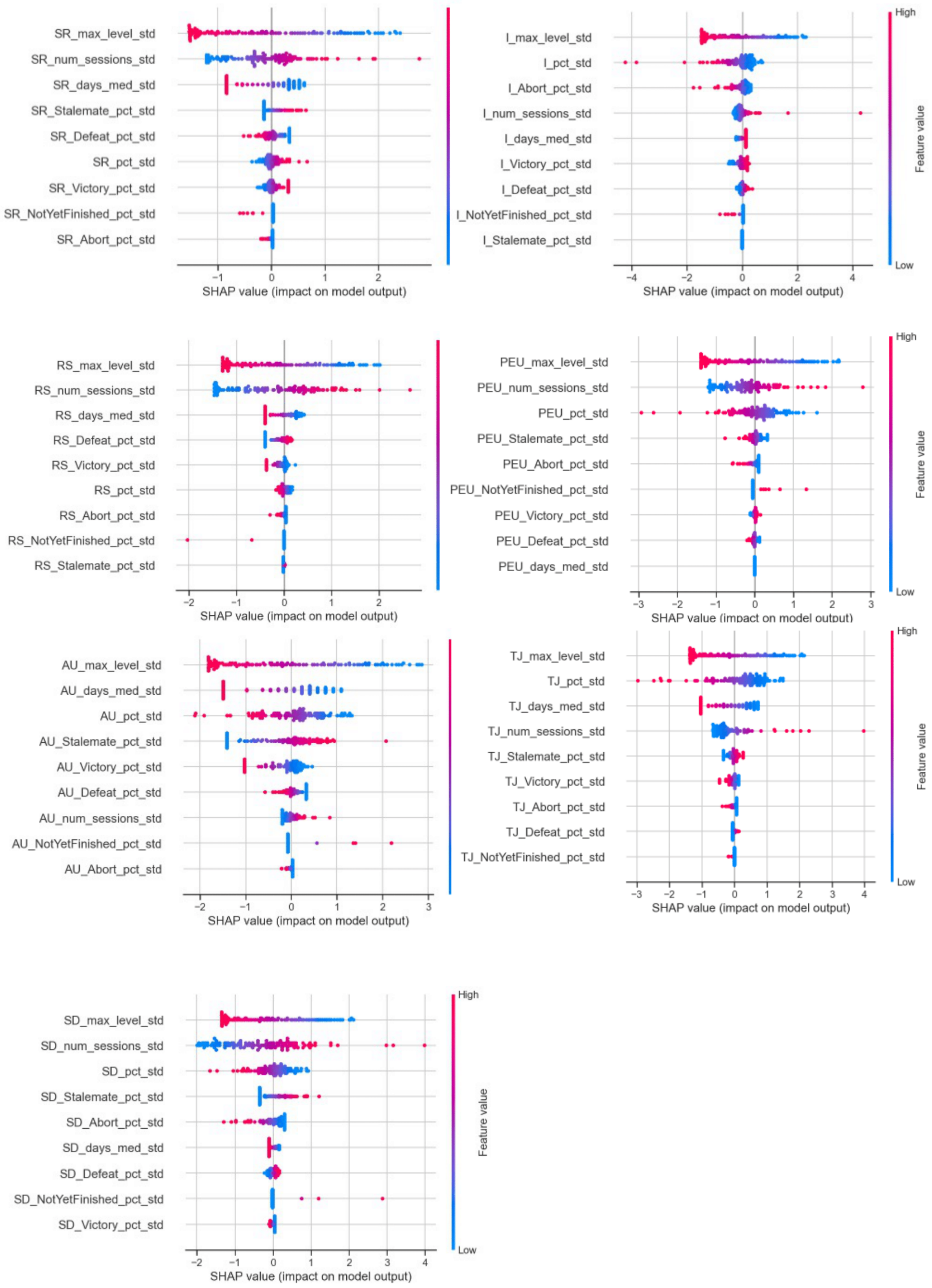

(b) Full Adherence.

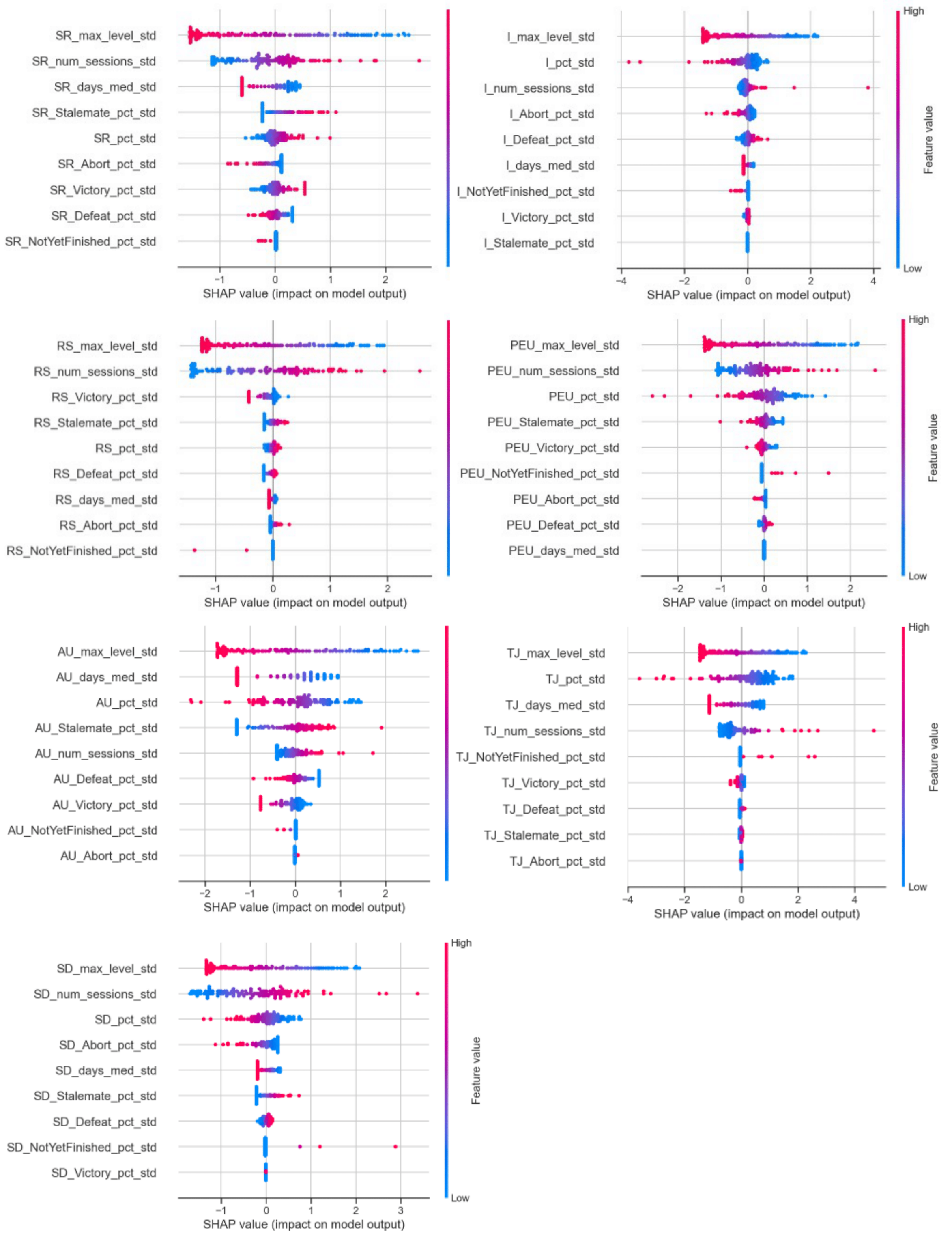

Supplement: S1 Fig — Evaluation of Game Performance Effects Using SHAP Values Summary Plot (a) Minimal Adherence (b) Full Adherence. (PDF) [file pone.0311279.s001.pdf]
