# Supplementary figures and images for: Predicting adherence to gamified cognitive training using early phase game performance data: Towards a just-in-time adherence promotion strategy
Source: PLoS One. 2024 Oct 2;19(10):e0311279. doi: 10.1371/journal.pone.0311279 (PMC11446454; doi:10.1371/journal.pone.0311279)

## Supporting Information

**S2 Fig. Weekly Fluctuations in Game Outcomes Over 12 Weeks**

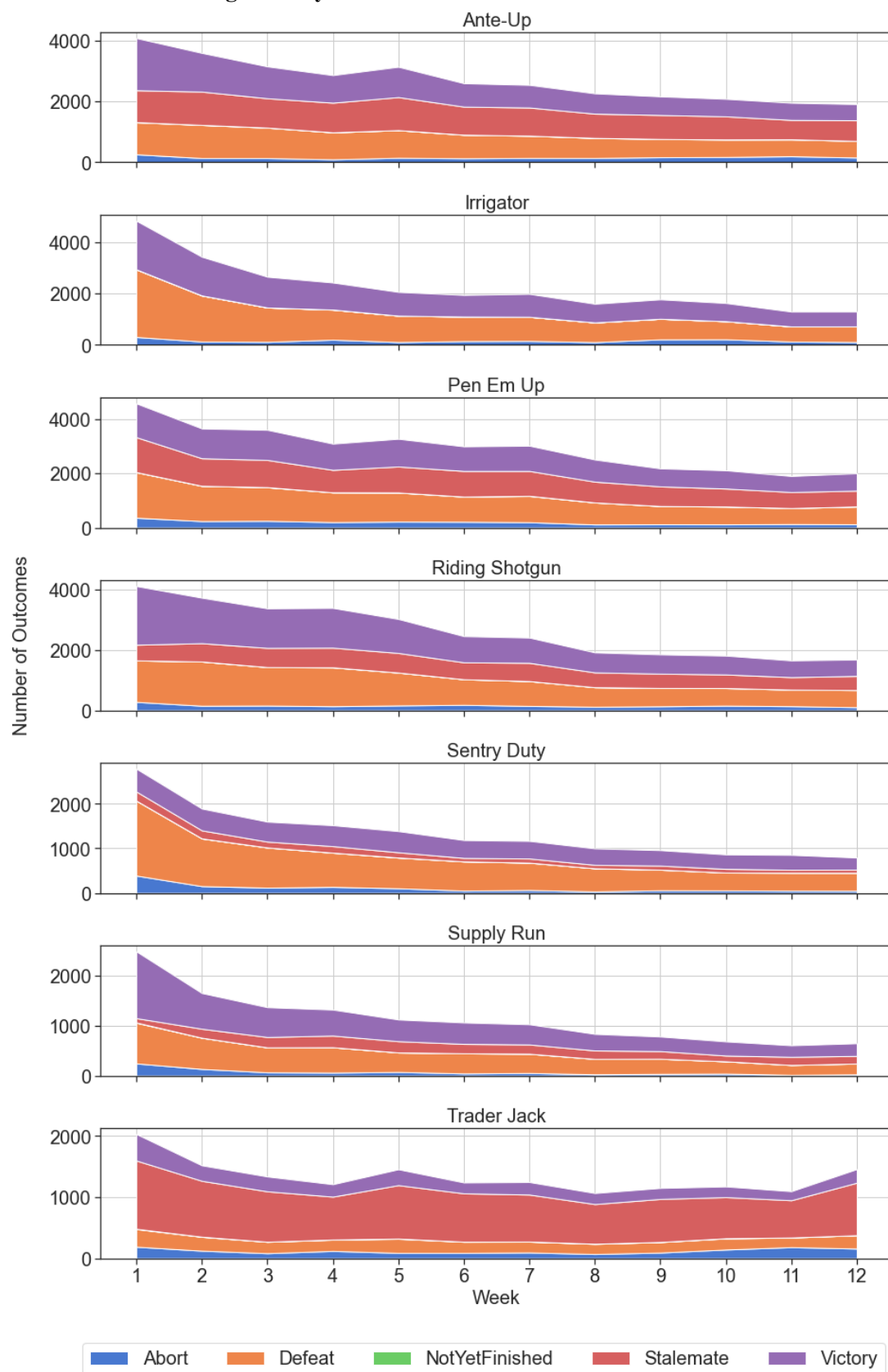

Supplement: S2 Fig — (PDF) [file pone.0311279.s002.pdf]
