# Supplementary material for: Predicting adherence to gamified cognitive training using early phase game performance data: Towards a just-in-time adherence promotion strategy
Source: PLoS One. 2024 Oct 2;19(10):e0311279. doi: 10.1371/journal.pone.0311279 (PMC11446454; doi:10.1371/journal.pone.0311279)
Supplement: S1 Table — (PDF) [file pone.0311279.s003.pdf]

Supporting Information

S3 Table. Descriptive Statistics of Attitude and Cognitive Scores for All Participants.

Gender		Mean (SD)
TSE Score	Female	14.5 (3.0)
	Male	15.5 (2.3)
	Unknown	16.5 (2.1)
	Total	14.9 (2.8)
GSE Score	Female	33.1 (3.5)
	Male	32.9 (3.4)
	Unknown	38.0 (1.4)
	Total	33.1 (3.5)
CPQ Score	Female	25.4 (4.8)
	Male	25.9 (4.9)
	Unknown	26.5 (0.7)
	Total	25.6 (4.8)
PDQ Score	Female	6.6 (2.7)
	Male	7.3 (2.8)
	Unknown	9 (7.1)
	Total	6.9 (2.8)
MDPQ Score	Female	26.7 (10.5)
	Male	27.7 (8.7)
	Unknown	30.8 (3.2)
	Total	27.1 (9.9)
MSEQ Score	Female	70.7 (16.2)
	Male	66.5 (18.3)
	Unknown	62.8 (5.3)
	Total	69.2 (12.9)
Technology Readiness Score	Female	12.9 (1.4)
	Male	12.9 (1.7)
	Unknown	13.3 (0.4)
	Total	12.9 (1.5)
NICT Score	Female	36.5 (5.3)
	Male	35.7 (4.1)
	Unknown	32.5 (2.1)
	Total	36.2 (5.0)
IADL Score	Female	0.8 (2.1)
	Male	0.4 (0.6)
	Unknown	2 (1.4)

	Total	0.7 (1.8)
Brain Training and Independence Score	Female	719.5 (597.0)
	Male	659 (672.3)
	Unknown	997.5 (116.7)
	Total	704.7 (616.4)
UFOV1 Score	Female	16.8 (8.0)
	Male	15.3 (1.4)
	Unknown	16.0 (1.6）
	Total	16.3 (6.6)
UFOV2 Score	Female	61.3 (79.8)
	Male	51.1 (61.3)
	Unknown	31.5 (23.5)
	Total	57.5 (63.6)
UFOV3 Score	Female	155.9 (83.9)
	Male	154.4 (102.6)
	Unknown	123.2 (26.0)
	Total	154.9 (89.4)
Raven's Advanced Progressive Matrices Score	Female	4.6 (2.7)
	Male	4.5 (2.2)
	Unknown	5.5 (2.1)
	Total	4.6 (2.5)
Digit Symbol Substitution Score 1	Female	45.7 (10.8)
	Male	41.6 (10.0)
	Unknown	40 (12.7)
	Total	44.3 (10.7)
Digit Symbol Substitution Score 2	Female	0.4 (2.1)
	Male	0.2 (0.6)
	Unknown	0 .0(0.0)
	Total	0.3 (1.7)
Digit Symbol Substitution Score 3	Female	45.3 (11.5)
	Male	41.3 (10.1)
	Unknown	40.0 (12.7)
	Total	43.9 (11.1)
Rey's AVLT Delayed recall Score	Female	7.9 (1.9)
	Male	6.1 (1.6)
	Unknown	8.0 (1.7)
	Total	7.3 (3.1)
Rey's AVLT Delayed Immediate Score	Female	53.3 (13.3)
	Male	45.1 (11.3)
	Unknown	50.5 (12.0)

	Total	50.6 (13.1)
Hopkins Delayed Recall Score	Female	9.0 (2.4)
	Male	7.8 (2.1)
	Unknown	6.5 (4.9)
	Total	8.6 (2.4)
Hopkins Immediate Recall Score	Female	6.5 (1.2)
	Male	5.9 (1.2)
	Unknown	5.5 (1.4)
	Total	6.3 (1.3)
