# Supplementary material for: Predicting adherence to gamified cognitive training using early phase game performance data: Towards a just-in-time adherence promotion strategy
Source: PLoS One. 2024 Oct 2;19(10):e0311279. doi: 10.1371/journal.pone.0311279 (PMC11446454; doi:10.1371/journal.pone.0311279)
Supplement: Attachment — Submitted filename: Response for Reviewer_PLOSONE_2_v3.pdf [file pone.0311279.s004.pdf]

Response for Reviewer's Comments

Thanks for the constructive feedback on our manuscript! We have revised the paper point-by-point as follows:

Reviewer #2: Thank you for the opportunity to re-review the article by Zhe He et al. While the reporting has improved and the aim and results are more comprehensible now, additional clarifications are required. I would also like to point out that while I understand the general idea behind the soft voting ensemble modelling technique now, I cannot judge whether it has been implemented and interpreted appropriately.

1) Thank you for clarifying the definition of adherence as a median split of the 10-week playing average and the way the binary outcome of the logistic regression is created. I understand that you are doing this split twice for different criteria, one at full adherence and once at minimal adherence which essentially yield 4 different subsamples which form the basis of all analyses. Please expand Table 1 and provide descriptive statistics for these 4 groups (age, sex, weekly adherence and the outcomes stated in Table 3).

Response: Thank you for the comments to provide me a new angle to make the descriptive statistics more completed. We revised the Table 1 as the below shows:

Table 1. Descriptive Statistics of the clinical trial data used in this study.

	Age<br>Mean (SD)	Gender<br>Count (Percent%)			Text Message Reminder Type<br>Count (Percent%)		
		Female	Male	Unknown	Negative-Framed	No Message	Positive-Framed
Minimal Adherence							
0	72.9(5.7)	38(48.7)	21(55.3)	1(50)	24	18	18
1	72.4(5.4)	40(51.3)	17(44.7)	1(50)	14	20	24
Full Adherence							
0	72.9(5.7)	39(50)	23(60.5)	1(50)	26	19	18
1	72.3(5.4)	39(50)	15(39.5)	1(50)	12	19	24

We also added adherence information in Table 3:

Table 3. Data obtained through gamified cognitive training interactions.

Category	Details
Text Message Reminder Type	Experimental condition assigned to participants: those receiving a positive message were coded as 1, those receiving a negative message were coded as -1, and those receiving no message were coded as 0.
Sessions	Sessions initiated by participants
Game levels	Levels for games with maximum levels ranging from 16 to 58 for different games

Game outcomes	Outcome of each game. Total 5 possible outcomes (Defeat, Stalemate, Victory, Abort, Not Yet Finished)
Number of sessions	The total number of sessions of different games participants played in previous two weeks
Percentage of games	The number of sessions for each game divides by the total number of sessions they played in previous two weeks
Percentage of outcomes	The number of sessions resulting in each specific outcome divided by the aggregate number of sessions conducted for each game in previous two weeks.
Maximum level	The highest-level participants reached in previous two weeks
Number of days reach middle level group	The number of days they spent on reaching median level for each game in previous two weeks
Minimal weekly adherence	The number of days they played at least 10 mins divided by 5
Full weekly adherence	The number of days they played at least 36 mins divided by 5
10-week minimal adherence	Average of minimal weekly adherence from week 3 to week 12
10-week full adherence	Average of full weekly adherence from week 3 to week 12
Minimal adherence level	If participants' 10-week minimal adherence value is greater than the median 10-week minimal adherence for all participants, it will be recorded as "1". Otherwise, it will be recorded as "0".
Full adherence level	If participants' 10-week full adherence value is greater than the median 10-week full adherence for all participants, it will be recorded as "1". Otherwise, it will be recorded as "0".

2) Relating to point 3 in my previous review report: the manuscript (L195) states that the median level is determined by the "highest levels reached" by all the participants and goes on to explain that if an individual passes the 25th percentile, they played further than 50% of other participants. Shouldn't this be the 50th percentile (the median?) and if not, could you please provide a clearer description of this procedure?

Response: Sorry for the confusion. To make it more understandable, we changed the variable name from "The number of days reaching median level" as "number of days reaching the middle level group". In defining the variable 'number of days reaching the middle level group,' we categorized participants' maximum levels from various games over the preceding two weeks into three distinct level group: low level group, middle level group, and high level group. These levels were determined using the 25th and 75th percentiles as thresholds. Specifically, scores falling within the 0-25th percentile were classified as "low level group", those within the 25th-75th percentile as "middle level groups", and scores exceeding the 75th percentile as "high level groups". I revised L184-L191 as:

"In defining the variable 'number of days reaching the middle level group,' we categorized participants' maximum levels from various games over the preceding two weeks into three

distinct level groups: low, middle, and high. These levels were determined using the 25th and 75th percentiles as thresholds. Specifically, levels falling within the 0-25th percentile were classified as “low level group”, those within the 25th-75th percentile as “middle level group”, and levels exceeding the 75th percentile as “high level group”. “Number of days reach middle level group” represents the number of days within a two-week span that a participant reaches this middle level group. If a participant does not achieve the middle level group within these two weeks, this variable is set to 15 days.”

3) Relating to point 4 in my previous review report: Table 4 does not show new columns with means and standard deviations. Did the authors provide the correct table?

Response: In our previous submission, we thought the composite score is not raw data, so we provided the means and standard deviations of raw data in S3. Based on your comments, we think it is also necessary to provide the means and standard deviations of composite scores. Thus, we revised the Table 4 as below:

Table 4. Calculation of Composite Scores Using Z-Scores.

Composite Score	Measurement	Mean (SD)
Composite score for technology proficiency	Average z-scores for the Computer Proficiency Questionnaire[20], Mobile Device Proficiency Questionnaire (MDPQ)[21]	2.619(0.772)
Composite score for self-efficacy	Averaging the z-scores of the General Self-efficacy Questionnaire (GSE)[22] and the Technology Self-efficacy Questionnaire (TSE) [22].	0.000(0.800)
Composite score for subjective cognition	Average z-score for the Instrumental Activities of Daily Living survey (IADL)[23], Perceived Deficits Questionnaire (PDQ)[24], and Memory Self-Efficacy Questionnaire (MSEQ)[25]	-0.010(0.644)
Composite score for perceived benefits	Average z-score for Brain Training (INDP)[26] and Independence Survey and Brain Training Belief Scale (NICT)[27]	0.063(0.710)
Composite score for objective reasoning	Average z-scores for Raven's Advanced Progressive Matrices[28,29] and Letter Sets[30].	-0.0133(0.863)
Composite score for objective processing speed	Average the z-scores for the Useful Field of View test (UFOV)[31] and Digit Symbol Substitution[32]	-0.010(0.644)
Composite score for objective memory immediate recall	Average z-score for Rey's Auditory Verbal Learning Test (Rey’s AVLT)[33] and Hopkins Verbal Learning Test (Hopkins memory test, immediate recall)[34]	0.068(0.816)

Composite score for objective memory delayed recall	Average z-score for Rey's AVLT delayed recall[33] and Hopkins Verbal Learning test (Hopkins memory test, delayed recall)[34]	0.007(0.831)

4) Relating to points 10 and 12 in my previous review report: despite the author's response, no changes were implemented in the manuscript. L215 continues to describe t-tests and chi-squared tests and the criterion of a p-value <= 0.1 for including them as predictors. However, Table 5 depicts these as "correlations". If I compute a chi-squared test on the provided contingency table for minimal adherence, then I get a non-1 p-value, which would suggest some (non-null) differences in the distribution of gender. The authors also comment on Cramer's V, which is not mentioned in the paper. I continue to be puzzled as to what Table 5 is actually showing. Furthermore, if gender indeed includes the stratum of 2 unknowns, then a chi-squared test cannot be used because the counts within the cells become too small. Fisher's exact test should be used in such a case.

Response: Sorry for the confusion. We have revised the table as p-values obtained from t-tests. Regarding the concern about applying chi-square tests to assess the correlation of gender and the adherence level, we decided to combine Male and Unknown in one category as "Male & Unknown". As such, we could use 2x2 contingency table with "Female" and "Male & Unknown" to get the p-value. We have revised the statement in L204-207 and the Table 5 as below:

"To assess the relationship between both continuous and categorical variables with adherence outcomes, t-tests were conducted for continuous variables to determine if their mean values differed significantly between participants who met the adherence threshold and those who did not. Additionally, Chi-square tests were applied to categorical variables to evaluate whether significant associations existed between the categorical variables and adherence. These analyses helped identify key predictors associated with adherence based on both continuous and categorical predictors. We chose those measures that showed statistical significance (alpha = 0.1) in the prediction models, and we ran separate analysis on both minimal level adherence and full level adherence to choose different sets of predictors. To mitigate the potential reduction in validity due to the small sample size in the "Unknown" gender category, we consolidated the gender variable into two groups: 'Female' and 'Male and Unknown.' This adjustment allowed us to apply the Chi-Square test."

Table 5. P-values between the variables and 10-week adherence at minimal and full levels

	p-value	
	Minimal Level	Full Level
Age	0.635	0.564
Gender	0.652	0.403
Test Message Reminder Type	0.169	**0.064**
Z-score for UFOV task	0.575	0.529
Z-score for digit symbol	0.774	0.606
Z-score for Ravens	0.970	0.659
Z-score for Letter sets	0.936	0.594

Z-score for Hopkins memory test, immediate recall	0.348	0.421
Z-score for Hopkins memory test, delayed recall	**0.037**	**0.039**
Z-score for Rey's AVLT, immediate recall	0.100	**0.060**
Z-score for Rey's AVLT, delayed recall	**0.030**	**0.014**
Z-score for IADL survey	0.975	0.936
Z-score for Brain Training Independence measure	0.804	0.764
Z-score for NICT	0.173	0.278
Z-score for MSEQ	0.587	0.699
Z-score for Perceived Deficits questionnaire (PDQ)	0.676	0.947
Z-score for General Self-efficacy (GSE)	**0.005**	**0.010**
Z-score for Technology Self-efficacy (TSE)	**0.054**	**0.068**
Z-score for Computer Proficiency Questionnaire (CPQ)	0.786	0.978
Z-score for Mobile Device Proficiency Questionnaire (MDPQ)	0.611	0.445
Z-score for tech readiness questionnaire	**0.055**	**0.084**
Composite score for technology proficiency	0.354	0.277
Composite score for self-efficacy	**0.003**	**0.006**
Composite score for IADL	0.547	0.406
Composite score for reasoning	0.703	0.339
Composite score for subjective cognition ability	0.643	0.842
Composite score for objective cognition ability, processing speed	0.605	0.485
Composite score for objective cognition ability, immediate recall	0.152	0.132
Composite score for objective cognition ability, delayed recall	**0.020**	**0.013**
Composite objective cognition ability, immediate and delayed recall	**0.047**	**0.036**

Minor:
5) It is much appreciated that the authors are sharing the code together with example data. The Notebook for the prediction models appears empty though - APPT_Feb16_24_ClassificationModels.ipynb has zero lines and 2 bytes file size.

Response: We don't know why it was a blank document. We have added the new one. Thank you for the reminder.

6) Table 1. "Std. of Age" is not the common abbreviation of standard deviation (SD). Please explain your abbreviation as part of the table caption/footnote.

Response: We have changed the Table 1 as I presented in previous answer. I changed "Std. of Age" as SD.

7) Table 5 and the line of text above are the only place referring to the variable "condition" - what is meant by this?

Response: The "condition" means that the message condition. During the cognitive training trial, the positive- and negative- framed messages about brain health were delivered to participants via the software program. There were two types of text messages. "1" means that they received positive-framed message (e.g. One of positive-framed message is "Regular metal challenge can have a positive impact on the brain"), "-1" means that they received negative-framed message, (e.g. One of negative-framed message is "Infrequent mental challenge can have a negative impact on brain")), and "0" means that

they received 0 message. We have changed the variable name to “Test Message Reminder Type” and added the description of this variable in Table 3.

To clarify it we added some statement in “The Cognitive Training Trail” part as below:

“The dataset used in this research was obtained from a previous clinical trial conducted on the Mind Frontiers mobile-based cognitive training game suite, designed explore how different message framings (positive-framed and negative-framed) about brain health influence adherence to a technology-based cognitive intervention and to identify individual differences that predict adherence. Florida State University Institutional Review Board approved the study protocol (IRB #: 2017.20622) and informed consent form. The recruitment period of this study was between July 1, 2017, and March 1, 2018. All the recruited participants provided written consent with their signatures. No minors were included in the study. The trial recruited 118 older adults living in the community, with a mean age of 72.6 years and a standard deviation of 5.54. Among these older adults, 78 are female (66%), 38 are male (32%), and 2 are unknown (2%) (see Table 1). Participants were randomly assigned to one of three groups: no message, positive-framed messages, or negative-framed messages (see Table 1). Those in the positive-framing group received messages emphasizing the benefits of engaging with cognitive training (e.g., " Regular metal challenge can have a positive impact on the brain "), while those in the negative-framing group received messages highlighting the risks of not engaging in such training (e.g., " Infrequent mental challenge can have a negative impact on brain "). Participants were instructed to engage in a cognitive training program consisting of seven games (see Table 2), with a target of five sessions per week, each lasting 45 minutes. The data collected included game levels achieved, ranging from 1 to 58, and five possible outcomes: Defeat, Stalemate, Victory, Abort, and Not Yet Finished. The dataset consists of two phases: in Phase 1, participants adhered to a structured 12-week program (45 minutes per day, 5 days per week), while in Phase 2, they were asked to play as frequently as desired during an unstructured 6-week period.”

8) Legibility of Table S3 would also benefit from rounding to one or two decimals and using a more common formatting such as “mean (SD)” within one cell.

Response: We revised it as below:

**Gender**		**Mean (SD)**
TSE Score	Female	14.5 (3.0)
	Male	15.5 (2.3)
	Unknown	16.5 (2.1)
	Total	14.9 (2.8)
GSE Score	Female	33.1 (3.5)
	Male	32.9 (3.4)
	Unknown	38.0 (1.4)

	Total	33.1 (3.5)
CPQ Score	Female	25.4 (4.8)
	Male	25.9 (4.9)
	Unknown	26.5 (0.7)
	Total	25.6 (4.8)
PDQ Score	Female	6.6 (2.7)
	Male	7.3 (2.8)
	Unknown	9 (7.1)
	Total	6.9 (2.8)
MDPQ Score	Female	26.7 (10.5)
	Male	27.7 (8.7)
	Unknown	30.8 (3.2)
	Total	27.1 (9.9)
MSEQ Score	Female	70.7 (16.2)
	Male	66.5 (18.3)
	Unknown	62.8 (5.3)
	Total	69.2 (12.9)
Technology Readiness Score	Female	12.9 (1.4)
	Male	12.9 (1.7)
	Unknown	13.3 (0.4)
	Total	12.9 (1.5)
NICT Score	Female	36.5 (5.3)
	Male	35.7 (4.1)
	Unknown	32.5 (2.1)
	Total	36.2 (5.0)
IADL Score	Female	0.8 (2.1)
	Male	0.4 (0.6)
	Unknown	2 (1.4)
	Total	0.7 (1.8)
Brain Training and Independence Score	Female	719.5 (597.0)
	Male	659 (672.3)
	Unknown	997.5 (116.7)
	Total	704.7 (616.4)
UFOV1 Score	Female	16.8 (8.0)
	Male	15.3 (1.4)
	Unknown	16.0 (1.6)
	Total	16.3 (6.6)
UFOV2 Score	Female	61.3 (79.8)
	Male	51.1 (61.3)
	Unknown	31.5 (23.5)

	Total	57.5 (63.6)
UFOV3 Score	Female	155.9 (83.9)
	Male	154.4 (102.6)
	Unknown	123.2 (26.0)
	Total	154.9 (89.4)
Raven's Advanced Progressive Matrices Score	Female	4.6 (2.7)
	Male	4.5 (2.2)
	Unknown	5.5 (2.1)
	Total	4.6 (2.5)
Digit Symbol Substitution Score 1	Female	45.7 (10.8)
	Male	41.6 (10.0)
	Unknown	40 (12.7)
	Total	44.3 (10.7)
Digit Symbol Substitution Score 2	Female	0.4 (2.1)
	Male	0.2 (0.6)
	Unknown	0 .0(0.0)
	Total	0.3 (1.7)
Digit Symbol Substitution Score 3	Female	45.3 (11.5)
	Male	41.3 (10.1)
	Unknown	40.0 (12.7)
	Total	43.9 (11.1)
Rey's AVLT Delayed recall Score	Female	7.9 (1.9)
	Male	6.1 (1.6)
	Unknown	8.0 (1.7)
	Total	7.3 (3.1)
Rey's AVLT Delayed Immediate Score	Female	53.3 (13.3)
	Male	45.1 (11.3)
	Unknown	50.5 (12.0)
	Total	50.6 (13.1)
Hopkins Delayed Recall Score	Female	9.0 (2.4)
	Male	7.8 (2.1)
	Unknown	6.5 (4.9)
	Total	8.6 (2.4)
Hopkins Immediate Recall Score	Female	6.5 (1.2)
	Male	5.9 (1.2)
	Unknown	5.5 (1.4)
	Total	6.3 (1.3)
